# Evaluation of Oxygenation in 129 Proning Sessions in 34 Mechanically Ventilated COVID-19 Patients

**DOI:** 10.1177/0885066620955137

**Published:** 2020-09-30

**Authors:** Max Berrill

**Affiliations:** 1 Department of Critical Care, 1333 St Peter’s Hospital, Surrey, United Kingdom

**Keywords:** Covid-19, prone positioning, ARDS

## Abstract

A minority of patients with Severe Acutre Respiratory Syndrome-Coronavirus-2 (SARS-CoV-2) disease-2019 (Covid-19) develop pulmonary features consistent with the Acute Respiratory Distress Syndrome (ARDS). Prone positioning (PP) is an intervention with proven survival benefits in moderate-to-severe and severe ARDS. It is advocated in international guidelines as an intervention in mechanically ventilated Covid-19 patients, despite very few published trials investigating its efficacy in Covid-19. There is an ongoing debate regarding the prevalence of reported mismatches between the severity of hypoxaemia and the preservation of pulmonary compliance in some patients, in the early stages of SARS-CoV-2 infection. This has led some to question its utility within this context. 129 proning sessions were identified in 34 consecutively prone patients admitted to the intensive care unit at a single center in the United Kingdom. Baseline characteristics of patients were consistent with previously published national and international reports and patients were ventilated in general concordance with the ARDSnet ventilation protocol. Paired analysis of the partial pressure of arterial oxygen(PaO_2_): fraction of inspired oxygen(FiO_2_) ratio (PF ratio) (n = 89) and FiO_2_ (n = 129) was recorded within 3 hours of both the initiation and termination of PP and differences were assessed with the paired Student’s *t*-test and Wilcoxon Signed-Rank test. Proning improved the PF ratio by 43.5 ± 54.9 from 99.8 ± 37.5 to 151.9 ± 58.9 (43.6% increase) [*p <* 0.0001] and reduced FiO_2_ by 0.17 ± 0.2 from 0.68 ± 0.2 to 0.51 ± 0.2 (25% decrease) [*p* < 0.0001]. 82% of proning maneouveres resulted in an improvement in the PF ratio. In summary, PP improved arterial oxygenation and reduced oxygen requirements in most Covid-19 patients in this single- center, retrospective analysis.

## Introduction

Severe Covid-19 displays similarities with “traditional” ARDS. It occurs rapidly within the context of respiratory symptoms without overt cardiac failure or fluid overload, bilateral pulmonary infiltrates are frequently discovered on radiographic imaging^1^ and arterial oxygenation is significantly impaired. Many patients experience refractory hypoxaemic respiratory failure despite mechanical ventilation. In contrast to non-Covid ARDS, however, at our center we have observed that pulmonary compliance is frequently maintained early in the course of disease progression. However, whether there is sufficient published evidence to support this as a consistent feature of Covid-19 remains contentious.^2^

Multiple randomized, controlled trials have indicated that arterial oxygenation improves with prone positioning compared to supine positioning in mechanically ventilated patients with ARDS.^3,4^ It improves mortality in a sub-population of patients with moderate-to-severe and severe ARDS when administered early, for sufficient periods of time^5^ and with appropriate ventilation strategies. It is suggested these effects in ARDS are mediated by reducing the ventral-dorsal transpulmonary pressure difference,^6^ homogenizing and stabilizing alveolar recruitment,^7^ reducing cardiac/diaphragmatic lung compression and improvement of ventilation/perfusion mismatch.

Prone positioning therefore has a plausible rationale for improving outcomes in Covid-19. It has been suggested as standard-of-care in international guidelines^8^ and has been reported to improve oxygenation.^2,9^ Despite these recommendations, there are currently very few published trials investigating changes in respiratory variables, such as arterial oxygenation, following proning in mechanically ventilated Covid-19 patients.

We therefore retrospectively assessed every proning session at our center during the initial 10 weeks of the Phase I Covid-19 pandemic to evaluate how patients responded in terms of arterial oxygenation.

## Methods

### Study Population

All patients studied were admitted to the Intensive Care Unit (ICU) at St Peter’s Hospital (SPH), Chertsey, Surrey, UK. SPH is a district general hospital which provides acute medical and surgical services for approximately 400,000 individuals in the south-west Greater London metropolitan area and the region westward. All patients admitted to the ICU with a provisional diagnosis of Covid-19 were assessed for whether they had been prone positioned.

### Proning Assessment

Patients were included for analysis if they were proned for >3 hours while mechanically ventilated and survived >24 hours from ITU admission (34/35 patients). Our center developed a policy of keeping patients supine if severely unstable and therefore those that did not survive >24 hours were likely to have been haemodynamically compromised and not considered for proning. The most recent arterial blood gas (ABG) measurements and ventilator settings were recorded within 3 hours of proning initiation or termination. If either the pre- or post- proning data was missing for one variable, both the pre- and post- data for that variable was omitted from analysis.

Ventilator settings are automatically updated to our center’s electronic record keeping systems and were therefore only rarely unavailable for analysis. ABGs, however, are taken at the discretion of the attending multi-disciplinary team and manually uploaded by critical care nursing staff. There are not mandated intervals in which ABGs are measured, therefore there was less PF ratio data from ABGs taken both 3 hours prior to initiation and termination of PP. In our center blood gas variables are recorded in kilo Pascals (kPa). These values were converted to mmHg by multiplying by 7.50062.

P_a_O_2_ data was not recorded as our center uses a titrated oxygenation targeting strategy in Covid-19 patients in which hypoxaemia is tolerated to a minimum threshold of 60 mmHg (8kPa). Many patients with severe respiratory failure therefore maintain P_a_O_2_ values of 60 mmHg (8kPa) with the attending team titrating the FiO_2_ to maintain this target. The PF ratio therefore provides a more insightful assessment of oxygenation within this context. The PEEP setting is chosen at the discretion of the attending team and is generally used in concordance with the ARDSnet guidance.

### Statisticual Analysis

All data, including patient characteristics, was manually recorded from Metavision^TM^ 5.46.44 (iMDsoft, Germany). Data was recorded in Microsoft Excel^TM^ (Microsoft, USA) and exported SPSS v26.0 (IBM, USA). Simple descriptive statistics presented in the tables are expressed as medians ± standard deviations and were evaluated in Excel^TM^ and comparison of paired respiratory system variables were performed using the paired Student *t-*test for parametic and Wilcoxon Signed-Rank test for non-parametric data in SPSS^TM^.

## Results

### Study Population

From 23^rd^ March 2020 to 7^th^ May 2020, 55 patients were admitted, of which 34 (61.8%) were proned for a total of 131 separate proning sessions. 33/34 of these patients had detectable SARS-CoV-2 RNA from nasopharyngeal swabs or bronchoalveolar lavage. The one patient without a detectable RNA sample was admitted to the ICU with clinical and radiographic features of Covid-19, had an initial negataive nasopharyngeal swab but died prior to a repeat sample being taken. Of the 131 separate prone sessions identified 89/131 (67.9%) and 129/131 (98.5%) proning sessions included either ABG or ventilator setting measurements, respectively, within 3 hours of both initiation and termination of prone positioning.

Characteristics of the patients are summarized in Table 1. 17/34 (50%) of patients died within 30 days of index admission.

**Table 1.** Characteristics of Patients (Median ± SD).

Characteristic	
Age—yr (n = 34)	58.5 ± 11.1
Male sex—no. (%) (n = 34)	29 (85.3)
BMI (n = 34)	31 ± 5.1
Admission Apache score (n = 32)	14 ± 4.7
Coexisting conditions—no. (%)	
Diabetes	13 (38.2)
* Hypertension*	15 (44.1)
* Lipid disorder*	9 (26.5)
* Chronic Kidney disease*	2 (5.8)
* Cardiovascular disease*	5 (14.7)
* Cancer*	3 (8.8)
* Respiratory disease*	8 (23.5)
* Immunodeficiency*	2 (5.8)

### Prone Position Characteristics

The characteristics of the prone positioning are displayed in Table 2. Patients were frequently proned within 2 days of admission. The average prone duration of each patient was 16.5 ± 2.7 hours, and patients were proned on average for 4 ± 2.4 separate proning sessions. Data on the time to ITU admission from hospital admission was missing for 2 individuals as these patients did not have a recorded time of hospital admission.

**Table 2.** Proning Characteristics (Median ± SD).

Time from hospital admission to ICU admission (hours) (n = 32)	42 ± 50.9
Time to first prone from ITU admission (hours) (n = 34)	23 ± 62.7
Number of times proned (n = 34)	4 ± 2.4
Total duration of proning (hours) (n = 34)	63.5 ± 38.2
Average duration of each prone (hours) (n = 34)	16.5 ± 2.7

### Mechanical Ventilation Characteristics

Table 3 shows the ventilator settings, respiratory mechanics and ABG measurements within 3 hours of when patients were first proned. Every patient during their first proning session was ventilated using either Pressure Regulated Volume Control (PRVC) or Airway Pressure Release Ventilation (APRV). PEEP settings were not recorded in patients ventilated in APRV (7/34). One patient ventilated with Pressure PRVC did not have PEEP settings available on the electronic record system. Patients were generally treated within the 6-8ml/kg IBW as recommended by the ARDSnet ventilator protocol, with an average TV/IBW of 7.86 ± 2.0 ml/kg at the initiation of the first proning session. Most patients met the Berlin criteria for severe ARDS with average PF ratios of 87.8 ± 38.2 and positive end-expiratory pressures of 10 ± 1.9 cm of H_2_O.

**Table 3.** Ventilatory Settings and Arterial Blood Gas Measurements at Start of First Proning Session (median ± SD).

Tidal Volume (ml) [n = 32]	525.5 ± 133.3
Tidal Volume (ml/kg of IBW) [n = 32]	7.86 ± 2.0
PEEP (cm of water) [n = 26]	10 ± 1.9
Respiratory frequency (breaths/min) [n = 33]	18 ± 4.2
Arterial pH [n = 32]	7.37 ± 0.1
PaCO2 (mmHg) [n = 32]	47.3 ± 8.9
Plasma bicarbonate (mmol/L) [n = 32]	24.6 ± 3.5
PaO_2_: FiO_2_ (mmHg) [n = 20]	87.8 ± 38.2
FiO_2_ [n = 34]	0.75 ± 0.19

### Response to Proning

Table 4 displays the baseline values of the measured oxygenation variables as well as the median of changes in oxygenation following a prone session. 72/89 (81%) of prone sessions resulted in an improvement in the PF ratio and the average absolute average change was 43.5 ± 54.9, representing a 43.6% increase. Proning sessions resulted in significantly reduced FiO2—by 0.17 ± 0.2 on average, which was a relative reduction of 25% from the baseline FiO2.

**Table 4.** Prone Responsiveness Measured by Arterial Oxygenation and Fraction of Inspired Oxygen (median ± SD).

		**Start**	**End**	**Change**	**% change**	**p value**
PaO_2_: FiO_2_ (n = 89) (mmHg)		99.8 ± 37.5	151.9 ± 58.9	43.5 ± 54.9	43.6	<0.0001*
FiO_2_ (n = 129)		0.68 ± 0.2	0.51 ± 0.2	-0.17 ± 0.2	-25.0	<0.0001**

* Paired Student’s t-test **Wilcoxon Signed-Rank test

## Discussion

Arterial oxygenation significantly improved in the majority of mechanically ventilated Covid-19 patients proned at our center. The majority of our patients were proned multiple times, using long prone-positioning sessions and within 24 hours of ICU admission. Generally, they were first proned in accordance with the ARDSnet protocol suggestion of 6-8ml/kg IBW tidal volumes with a PEEP >5 cm H_2_O (or PEEP of 10 cm H_2_O if FiO_2_ is between 0.7-0.8, as our patients averaged). These results are relatively consistent with methodology in both the randomized controlled trials and subsequent meta-analyses in non-Covid related ARDS^10^ and the anecdotal evidence from other centers treating this emergent global pandemic.

There continues to be uncertainty as to whether Covid-19 hypoxaemic respiratory failure is an extension of the previously recognized ARDS, whether it represents alternative phenoytpes of this heterogenous syndrome^11^ or whether it represents a novel disease with a more normal distribution of pulmonary compliance than would be suggested by phenotyping.^12^ This could arguably create uncertainty as to whether interventions in traditional ARDS will therefore be effective in “Covid-19 ARDS.” In particular, suggestions that in some patients pulmonary compliance is well-maintained in Covid-19 lung injury^13^ suggests an alterative pathophysiology. This may be related to the significant prothrombotic features which characterize an important aspect of this novel disease.

In ARDS, the repositioning of proning improves the efficiency of ventilation-perfusion matching by recruiting densely populated alveolar units in the dorsal aspect of the lung. This recruitment is driven by improved compliance secondary to reduced compression by the abdomen and the heart.^14^ These results suggest that the Covid lung remains responsive to prone-positioning, which is likely due to the mechanisms which improve oxygenation in ARDS. The thrombosis which appears characteristic of Covid-19 was a likely contributor to perfusion redistribution in the patients in this study and the interstitial oedema evidenced by infiltrates on radiographic imaging will also have contributed to V/Q changes. Patients generally presented with dry coughs and focal consolidation on chest radiographs was not a common early feature at our center. It is therefore unlikely that bacterial superinfection was a major contributing factor as these patients were generally proned early in the course of their admission.

As a collective term, ARDS already encompasses a constellation of differing pathophysiologies and these results suggest that Covid-19 may share similar features of oxygenation impairment due to alveolar derecuitment, pulmonary shunting and increased dead space. As the patients in this study responded beneficially to the V/Q changes of proning these results indicate that despite a novel pathophysiological process, severe Covid-19 lung injury retains similar features to other acute pulmonary conditions.

In conclusion, this retrospective analysis indicated that patients with ARDS in the context of Covid-19 and hypoxaemia, prone positioning improved oxygenation. Further studies, including prospective studies and controlled trials, into the relationship the impact of prone positioning on mortality are therefore warranted.

## References

[1] Shi H Han X Jiang N, et al. Radiological findings from 81 patients with COVID-19 pneumonia in Wuhan, China: a descriptive study. Lancet Infect Dis. 2020;20(4):425–434. 32105637 10.1016/S1473-3099(20)30086-4 PMC7159053

[2] Ziehr DR Alladina J Petri CR, et al. Respiratory pathophysiology of mechanically ventilated patients with COVID-19: a cohort study. Am J Respir Crit Care Med. 2020;201(12):1560–1564. 32348678 10.1164/rccm.202004-1163LE PMC7301734

[3] Munshi L Del Sorbo L Adhikari NK, et al. Prone position for acute respiratory distress syndrome. A systematic review and meta-analysis. Ann Am Thorac Soc. 2017;14(suppl 4):S280–S288. 29068269 10.1513/AnnalsATS.201704-343OT

[4] Sud S Friedrich JO Taccone P, et al. Prone ventilation reduces mortality in patients with acute respiratory failure and severe hypoxemia: systematic review and meta-analysis. Intensive Care Med. 2010;36(4):585–599. 20130832 10.1007/s00134-009-1748-1

[5] Guérin C Reignier J Richard JC, et al. Prone positioning in severe acute respiratory distress syndrome. N Engl J Med. 2013;368(23):2159–2168. 23688302 10.1056/NEJMoa1214103

[6] Pelosi P Brazzi L Gattinoni L Prone position in acute respiratory distress syndrome. Eur Respir J. 2002;20(4):1017–1028. 12412699 10.1183/09031936.02.00401702

[7] Cornejo RA Díaz JC Tobar EA, et al. Effects of prone positioning on lung protection in patients with acute respiratory distress syndrome. Am J Respir Crit Care Med. 2013;188(4):440–448. 23348974 10.1164/rccm.201207-1279OC

[8] Alhazzani W Møller MH Arabi YM, et al. Surviving sepsis campaign: guidelines on the management of critically ill adults with coronavirus disease 2019 (COVID-19). Crit Care Med. 2020;48(6):e440–e469. 32224769 10.1097/CCM.0000000000004363 PMC7176264

[9] Pan C Chen L Lu C, et al. Lung recruitability in COVID-19–associated acute respiratory distress syndrome: a single-center observational study. Am J Respir Crit Care Med. 2020;201(10):1294–1297. 32200645 10.1164/rccm.202003-0527LE PMC7233342

[10] Alsaghir AH Martin CM Effect of prone positioning in patients with acute respiratory distress syndrome: a meta-analysis. Crit Care Med. 2008;36(2):603–609. 18216609 10.1097/01.CCM.0000299739.98236.05

[11] Gattinoni L Chiumello D Caironi P, et al. COVID-19 pneumonia: different respiratory treatments for different phenotypes? Intensive Care Med. 2020;46(6):1099–1102. 32291463 10.1007/s00134-020-06033-2 PMC7154064

[12] Bos LDJ Sinha P Dickson RP The perils of premature phenotyping in COVID: a call for caution. Eur Respir J. 2020;56(1):2001768. 32482788 10.1183/13993003.01768-2020 PMC7263061

[13] Gattinoni L Coppola S Cressoni M Busana M Rossi S Chiumello D Covid-19 does not lead to a “typical” acute respiratory distress syndrome. Am J Respir Crit Care Med. 2020;201(10):1299–1300. 32228035 10.1164/rccm.202003-0817LE PMC7233352

[14] Albert RK Hubmayr RD The prone position eliminates compression of the lungs by the heart. Am J Respir Crit Care Med. 2000;161(5):1660–1665. 10806172 10.1164/ajrccm.161.5.9901037

